# Supplementary material for: Validation of color Doppler ultrasound and computed tomography in the radiologic assessment of non-malignant acute splanchnic vein thrombosis
Source: PLoS One. 2021 Dec 20;16(12):e0261499. doi: 10.1371/journal.pone.0261499 (PMC8687587; doi:10.1371/journal.pone.0261499)
Supplement: S1 Table — (DOCX) [file pone.0261499.s001.docx]

**S1 Table. Overview of studies investigating imaging techniques in the diagnosis of acute splanchnic vein thrombosis**

| **Study** | **Patients** | **Imaging techniques investigated** | **Gold standard** | **Remarks** |
| --- | --- | --- | --- | --- |
| Miller et al. (1985)^1^ | 5 | CDUS vs. CT | - |  |
| Tessler et al. (1991)^2^ | 75 (9) | CDUS | angiography (n = 13), surgery (n = 62) | 9 patients had PVT |
| Haddad et al. (1992)^3^ | 6 CT  3 CDUS | CDUS vs. CT vs. MRI | - |  |
| Tanaka et al. (1993)^4^ | 18 | CDUS vs. angiography | autopsy/surgery | malignant PVT (HCC) |
| Bach et al. (1996)^5^ | 63 (41) | CDUS vs. CTAP | surgery | 41 patients had PVT,  malignant PVT |
| Rossi et al.  (2006)^6^ | 316 (79) | CEUS vs. CDUS | Biopsy/CT/MRI | 79 patients had PVT,  malignant PVT |
| Rossi et al. (2008)^7^ | 50 | CEUS vs. CT | biopsy | malignant PVT (HCC) |

Abbreviations: CDUS = color Doppler ultrasound, CEUS = contrast-enhanced ultrasound, CT = computed tomography, CTAP = computed tomography with arterial spleno-portography, HCC = hepatocellular carcinoma, MRI = magnetic resonance imaging, PVT = portal vein thrombosis

References:

1. Miller VE, Berland LL. Pulsed Doppler duplex sonography and CT of portal vein thrombosis. *Am J Roentgenol*. 1985;145(1):73-76. doi:10.2214/ajr.145.1.73

2. Tessler FN, Gehring BJ, Gomes AS, et al. Diagnosis of portal vein thrombosis: value of color Doppler imaging. *Am J Roentgenol*. 1991;157(2):293-296. doi:10.2214/ajr.157.2.1853809

3. Haddad MC, Clark DC, Sharif HS, al Shahed M, Aideyan O, Sammak BM. MR, CT, and ultrasonography of splanchnic venous thrombosis. *Gastrointest Radiol*. 1992;17(1):34-40. http://www.ncbi.nlm.nih.gov/pubmed/1544556.

4. Tanaka K, Numata K, Okazaki H, Nakamura S, Inoue S, Takamura Y. Diagnosis of portal vein thrombosis in patients with hepatocellular carcinoma: efficacy of color Doppler sonography compared with angiography. *Am J Roentgenol*. 1993;160(6):1279-1283. doi:10.2214/ajr.160.6.8388620

5. Bach AM, Hann LE, Brown KT, et al. Portal vein evaluation with US: comparison to angiography combined with CT arterial portography. *Radiology*. 1996;201(1):149-154. doi:10.1148/radiology.201.1.8816536

6. Rossi S, Rosa L, Ravetta V, et al. Contrast-enhanced versus conventional and color doppler sonography for the detection of thrombosis of the portal and hepatic venous systems. *Am J Roentgenol*. 2006;186(3):763-773. doi:10.2214/AJR.04.1218

7. Rossi S, Ghittoni G, Ravetta V, et al. Contrast-enhanced ultrasonography and spiral computed tomography in the detection and characterization of portal vein thrombosis complicating hepatocellular carcinoma. *Eur Radiol*. 2008;18(8):1749-1756. doi:10.1007/s00330-008-0931-z

8. Landis JR, Koch GG. The Measurement of Observer Agreement for Categorical Data. *Biometrics*. 1977;33(1):159. doi:10.2307/2529310
