# Supplementary material for: Validation of color Doppler ultrasound and computed tomography in the radiologic assessment of non-malignant acute splanchnic vein thrombosis
Source: PLoS One. 2021 Dec 20;16(12):e0261499. doi: 10.1371/journal.pone.0261499 (PMC8687587; doi:10.1371/journal.pone.0261499)
Supplement: S2 Table — (DOCX) [file pone.0261499.s002.docx]

**S2 Table. Classification of Cohen’s kappa according to Landis & Koch^1^**

| **Cohen’s kappa** | **Strength of agreement** |
| --- | --- |
| < 0 | poor |
| 0.00-0.20 | slight |
| 0.21-0.40 | fair |
| 0.41-0.60 | moderate |
| 0.61-0.80 | substantial |
| 0.81-1.00 | almost perfect |

Reference:

1. Landis JR, Koch GG. The Measurement of Observer Agreement for Categorical Data. *Biometrics*. 1977;33(1):159. doi:10.2307/2529310
