# Supplementary material for: Validation of color Doppler ultrasound and computed tomography in the radiologic assessment of non-malignant acute splanchnic vein thrombosis
Source: PLoS One. 2021 Dec 20;16(12):e0261499. doi: 10.1371/journal.pone.0261499 (PMC8687587; doi:10.1371/journal.pone.0261499)
Supplement: S3 Table — (DOCX) [file pone.0261499.s003.docx]

**S3 Table. Ultrasound and CT devices utilized at the study centers**

| **University Medical Center Freiburg** |
| --- |
| **Ultrasound** Philips EPIQ 5 (Philips Health Systems, Hamburg, Germany**)**  Siemens Acuson S 2000 (Siemens Healthcare GmbH, Erlangen, Germany)  Esaote MyLab (Esaote SpA, Genoa, Italy)  Toshiba Aplio MX (Toshiba Medical Systems Corporation, Ōtawara, Japan)  **CT** Siemens Somatom Definition Flash (Siemens Healthcare GmbH, Erlangen, Germany)  Toshiba Aquilion One (Toshiba Medical Systems Corporation, Ōtawara, Japan) |
| **RKH Hospital Ludwigsburg** |
| **Ultrasound** Siemens Acuson S 3000 (Siemens Healthcare GmbH, Erlangen, Germany)  Philips iU22 (Philips Health Systems, Hamburg, Germany**)**  Hitachi Ascendus (Hitachi Medical Corporation, Tokyo, Japan)  GE Logiq P5 (GE Healthcare, Chalfont St. Giles, United Kingdom)  Siemens Sequoia 512 (Siemens Healthcare GmbH, Erlangen, Germany)  **CT** Siemens Somatom Definition Flash (Siemens Healthcare GmbH, Erlangen, Germany)  Siemens Somatom Definition AS (Siemens Healthcare GmbH, Erlangen, Germany) |
