# Supplementary material for: Validation of color Doppler ultrasound and computed tomography in the radiologic assessment of non-malignant acute splanchnic vein thrombosis
Source: PLoS One. 2021 Dec 20;16(12):e0261499. doi: 10.1371/journal.pone.0261499 (PMC8687587; doi:10.1371/journal.pone.0261499)
Supplement: Attachment — Submitted filename: RESPONSE TO REVIEWERS.docx [file pone.0261499.s005.docx]

**Point-by-point response to academic editor’s remarks**

**1. Please ensure that your manuscript meets PLOS ONE's style requirements, including those for file naming.**

We apologize for any deviations from PLOS ONE’s style requirements. We have revised the manuscript in this respect.

**2. In your ethics statement in the Methods section and in the online submission form, please provide additional information about the data used in your retrospective study. Specifically, please ensure that you have discussed whether all data were fully anonymized before you accessed them and/or whether the IRB or ethics committee waived the requirement for informed consent. If patients provided informed written consent to have data from their medical records used in research, please include this information.**

**3. Please include the date(s) on which you accessed the databases or records to obtain the data used in your study.**

Thank you for this hint. We have added this information in the ‘materiales and methods‘ section.

*“The multi-center study was approved by the local ethics committee (no. EK 85/19) and is in accordance with the Declaration of Helsinki. Due to the retrospective design of the study the requirement for informed patient consent was waived. Patient records were accessed between 1^st^ January and 31^st^ January 2020 to obtain the study data. All patient data were pseudonymized. The study was registered in the German Clinical Trials Register (DRKS 00019008).”*

**4. In your Data Availability statement, you have not specified where ALL the minimal data set underlying the results described in your manuscript can be found. PLOS defines a study's minimal data set as the underlying data used to reach the conclusions drawn in the manuscript and any additional data required to replicate the reported study findings in their entirety. All PLOS journals require that the minimal data set be made fully available.**

We have now added ‘S1 File‘ which contains the study data set as supporting material.

**5. Thank you for stating the following in the Acknowledgments Section of your manuscript: [LS and DB aresupported by the Berta-Ottenstein-Programme, Faculty of Medicine, University of Freiburg.] We note that you have provided funding information that is not currently declared in your Funding Statement. However, funding information should not appear in the Acknowledgments section or other areas of your manuscript. We will only publish funding information present in the Funding Statement section of the online submission form. Please remove any funding-related text from the manuscript and let us know how you would like to update your Funding Statement. Currently, your Funding Statement reads as follows: [The authors received no specific funding for this work.] Please include your amended statements within your cover letter; we will change the online submission form on your behalf.**

The Berta-Ottenstein-Programme is an ‘in-house‘ excellency program of the Faculty of Medicine, University of Freiburg. This affiliation is usually not listed as funding in publications. However, if PLOS ONE’s policy considers it as funding, we have absolutely no objections against removing the Acknowledgments section of our manuscript and stating „LS and DB are supported by the Berta-Ottenstein-Programme, Faculty of Medicine, University of Freiburg.” as funding information instead.

**6. Please include captions for your Supporting Information files at the end of your manuscript, and update any in-text citations to match accordingly.**

We have gladly amended captions for S1-S3 Tables as well as S1 File.

**Point-by-point response to reviewers’ remarks**

**Reviewer #1**

**1.Is CT more reliable than CDUS if the evaluator is not an experienced interventional hepatologists/radiologists?**

Thank you very much for pointing out this aspect. Indeed, training level of the investigator may significantly impact imaging results, especially with regard to CDUS. This aspect is discussed in the revised version of our manuscript.

*“Importantly, our results may not be fully transferable to all clinical settings, as the investigator’s experience may influence imaging results, especially in the case of CDUS.”*

**2.Were the CT and CDUS purchased at about the same time and with the same high performance? Is the CT the old one and the US the newest one?**

Due to the study design a number of different CDUS and CT devices were used at the two study centers. However, as tertiary care hospitals, both centers were equipped with modern imaging devices of comparable technical standard. We have listed CDUS and CT devices as Supporting Table (S3 Table).

**Reviewer #2**

**Major**
**#1. The number of enrollment was only 49 patients. I think this is too small.**

We absolutely agree that our patient collective is relatively small for a retrospective observational analysis. As acute splanchnic thrombosis is a very rare condition, recruiting larger patient collectives is challenging. Hence, realizing that previous imaging studies on splachnic thrombosis (summarized in ‘S1 Table‘) investigated comparable patient numbers, we hope that our results still have a certain meaningfulness, as they provide data in a field that is only very poorly studied until now. Naturally, our results require validation in further studies with larger patient collectives. We have included this aspect in the ‘Discussion‘ and ‘Conclusions‘ sections of our manuscript in order to point out the limitations coming along with a relatively small patient collective.

*“As acute splanchnic vein thrombosis is a very rare condition with an incidence of < 1/100.000 per year we chose a retrospective study design, which allowed us to include 49 patients in our analysis [19]. This patient collective size is comparable with previous imaging studies on splanchnic thrombosis [12-18]. Still, it is important to keep the limited number of patients in mind when interpreting the significance our findings.”*

*“Naturally, our results require validation in further studies of larger patient collectives.”*

**#2. In clinical, CDUS and enhanced-CT are useful and often performed for diagnosis of portal or splanchnic vein thrombosis. We often perform direct transjugular spleno-portography after being diagnosed in order to intervention therapy. So, this conclusion seems to be low impact.**

Thank you very much for this remark. Our clinical experience matches yours – in most cases patients are allocated to portography (plus interventional therapy) after diagnosis of acute splachnic thrombosis by CDUS or CT. Indeed, all patients in our study received portography following CDUS and/or CT. The rationale of our study was to compare the reliabilty of CDUS and CT in grading splachnic thrombosis, as this may help clinicians to interpret the significance of the findings of CDUS and CT, for example when making the decision for or against allocating patients to interventional therapy of thrombosis.

**#3. Concerns to patient characters, 35 patients have non-cirrhotic thrombosis. What were their detail cause? What number was due to surgery?**

‘Table 1‘ now lists the different etiologies of non-cirrhotic thrombosis. Indeed, only two patients in our collective had to be transferred to surgery. This may be due to patient selection as we included patients primarily allocated to portography (and interventional therapy) after being diagnosed with acute splachnic thrombosis, while patients requiring surgery may have been directly transfered to surgical treatment.

**Table 1. Patient characteristics**

| Characteristics | Number |
| --- | --- |
| Patients total | 49 |
| Mean age [years] | 56 ±13.5 |
| Gender  male  female | 29 (59.2%)  20 (40.8%) |
| Etiology  cirrhotic thrombosis  non-cirrhotic thrombosis  myeloproliferative disease  thrombophilia  infective/inflammatory disease  after abdominal surgery  idiopathic | 14 (28.6%)  35 (71.4%)  8 (16.3%)  6 (12.2%)  6 (12.2%)  2 (40.8%)  13 (26.5%) |
| Portography + CDUS *+* CT | 26 (53.1%) |
| Portography + CDUS | 8 (16.3%) |
| Portography + CT | 15 (30.6%) |
| Time CDUS/CT to portography* [days] | 1.95 ±2.38 |

* The time span to spleno-portography was calculated from the date of CDUS or CT depending on which imaging procedure was performed first

Abbreviations: CDUS = color Doppler ultrasound, CT = contrast-enhanced computed tomography

Minor
#1. Images of CDUS and CT as figures should be added.

Thank you for this suggestion. We have added an example of CDUS, CT and portography as ‘Fig 4‘.

**Fig 4: Example of divergent findings in CDUS and CT**

While the main trunk of the portal vein was graded as ‘partial thrombosis’ by CDUS (A), since thrombotic material could be identified in the vessel lumen but a preserved portal venous flow signal was detectable, it was graded as ‘complete thrombosis’ by CT (B) and portography (C), as both showed thrombotic occlusion of the vessel lumen without contrast agent flow.

**Reviewer #3:** 

**Please consider a subanalysis comparing the 26 patients who had all 3 imaging modalities.**

Thank you very much for suggesting this sensible sub-analysis. It revealed that – while both performed slightly worse than in the whole patient collective – CDUS and CT still showed almost identical rates of consistency with portographic results:

*“In a sub-analysis of the 26 patients in which both CDUS and CT were available in addition to portography, CDUS and CT displayed lower consistency with portography. Still, both imaging techniques performed almost equally well (66.9% vs. 68.5% consistency).”*

**Compare CT and US versus portography and also US vs CT. This also may be of value as CT and US are used interchangeably when they may be not.**

Again, thank you for this recommendation. Comparison of CDUS and CT revealed that their gradings of thrombosis matched in 74.4 % of cases, indicating that the two imaging modalities cannot fully be used interchangeably, which undoubtfully is a relevant result that we added to the manuscript:

*“The gradings of thrombosis obtained by CDUS and CT matched in 74.4 % of cases, indicating that despite their similar performance the results of the two imaging techniques were not fully interchangeable.”*

**It is possible that among those patients in whom there was a discrepancy between portography and the other imaging modalities the time lapse between imaging modalities might have been longer (i.e. the CDUS or CT were done first – failing to detect a thrombus, that subsequently developed and was later found by portography). Please clarify this.**

To investigate whether the period of time between initial diagnosis of splachnic thrombosis by CDUS/CT and portographic assessment of thrombosis had any influence on the performance of CDUS and CT, we separately analzyed patients with a time span of less than 48 hours and 48 hours or more between CDUS/CT and portography. However, we observed no significant difference in consistency of CDUS/CT with portographic findings between the two patient groups.

*“We assessed if the time span between initial detection of splanchnic thrombosis by CDUS/CT and portography influenced the performance of the imaging techniques. However, consistency with portographic findings was not significantly different between patients with a time span of less than 48 hours and patients with 48 hours or more between CDUS/CT and portography (p ≥ 0.296).”*

**Please make sure that the study adheres to STROBE guidelines.**

We have checked that the study adheres to the STROBE guidelines and have added a remark in the ‘Materials and methods‘ section.

**Figure 2 could use a single panel with 5 100%-stacked columns, one per vessel. Would make it much clearer.**

Thank you for this sensible suggestion. We have changed ‘Fig 2‘ accordingly.

**The manuscript needs a thorough revision of the English language.**

We sincerely apologize for any linguistic errors. We have revised grammar and orthography.
